# The comparison between the novel technique and conventional method in the catheter ablation of premature ventricular contractions originating from the free wall of tricuspid annulus

**DOI:** 10.1002/clc.24179

**Published:** 2023-10-25

**Authors:** Mingxian Chen, Songyun Wang, Tongjian Zhu, Xuping Li, Zhihong Wu, Qiming Liu, Shenghua Zhou

**Affiliations:** ^1^ Department of Cardiology The Second Xiangya Hospital of Central South University The Second Xiangya Hospital of Central South University Changsha Hunan People's Republic of China; ^2^ Department of Cardiology Wuhan Renmin Hospital of Wuhan University Wuhan China; ^3^ Department of Cardiology Xiangyang Central Hospital Xiangyang Hubei China

**Keywords:** ablation, free wall, premature ventricular contraction, reversed S‐curve technique, steerable sheath, tricuspid annulus

## Abstract

**Background:**

This study aimed to assess the safety and effectiveness of a novel technique for catheter ablation in patients with premature ventricular contraction (PVC) from the free wall of tricuspid annulus (TV).

**Hypothesis:**

We hypothesized that the novel technique is more efficacious than the traditional approach.

**Methods:**

We retrospectively investigated 59 consecutive patients with PVC originating from the free wall of TV between January 2013 and November 2021. The patients were divided into two groups: the reversed S‐curve technique group (RST, *n* = 26) and the reversed C‐curve technique group (RCT, *n* = 33). The RST under the support of a steerable sheath was used in RST group, while the RCT under the support of a nonsteerable sheath was used in the RCT group. Systematic mapping and radiofrequency ablation were preferentially performed under the valve in all patients.

**Results:**

Compared to the RCT group, total procedural time and fluoroscopic exposure time were significantly shorter in RST group. Two patients experienced cardiac tamponade in the RCT group, while no complications were observed in RST group (*p* = .498). The success rate was significantly higher in RST group compared to RCT group (81.9% vs. 100%, *p* = .029). Three patients in RCT group failed to ablate during the operation but were successfully ablated using the novel method. During regular follow‐up, no patients in the RST group had a recurrence, while three patients in the RCT group did (*p* = .274).

**Conclusions:**

It suggests that the reserved S‐curve technique, supported by a steerable sheath, is a feasible and effective method for ablating PVC originating from the free wall of TV.

## Graphical abstract

This study focus on the safety and effectiveness of the reversed S‐curve technique under the support of a steerable sheath for catheter ablation in patients with premature ventricular contraction (PVC) from free wall of tricuspid annulus (TV). The data showed that the reserved S‐curve technique, supported by a steerable sheath, is a feasible and effective method for ablating PVC originating from the free wall of TV.

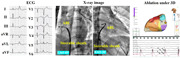

## 1 INTRODUCTION

Although idiopathic premature ventricular contractions (PVC) are usually not life‐threatening, they are often symptomatic and cause arrhythmic cardiomyopathy. Idiopathic PVCs originate from specific anatomical structures according to the anatomical background. Idiopathic PVCs are commonly originating from the right ventricular outflow tract, with a small part of them originating from the left ventricular outflow tract.^1^, ^2^, ^3^ In recent years, a small number of cases of idiopathic PVCs have been proven to derive from some uncommon sites of idiopathic PVCs such as tricuspid annulus (TA).^4^, ^5^ However, it was not so effective for the radiofrequency ablation of PVCs originating from free wall of TA.^6^ The most likely reason for the failure cases was an inadequate burn due to the poor contact and the ablation catheter instability.^7^ Catheter ablation of PVCs deriving from the free wall also has a risk of right ventricular perforation. Therefore, catheter ablation of PVC originating from free wall of tricuspid annulus (TV) represents a challenging task and the success rate is lower than the other regions of TV. The purpose of this study was to investigate the safety and efficacy of a novel technique in catheter ablation for PVC from free wall of TV via a reversed S‐curve technique (RST) under the support of a steerable sheath.

## 2 MATERIALS AND METHODS

### 2.1 Study population

In this retrospective study, 59 patients underwent radiofrequency ablation for idiopathic PVCs originating from the free wall of the TA at the Catheter Center of The Second Xiangya Hospital of Central South University, Wuhan Renmin Hospital, and Xiangyang Central Hospital between January 2013 and November 2021. Patients were randomly assigned to two groups: the RST group (*n* = 26), in which the RST was used with the support of a steerable sheath, and the reversed C‐curve technique (RCT) group (*n* = 33), in which the RCT was used with the support of a nonsteerable sheath. Various electrophysiological properties of idiopathic PVCs originating from the free wall of TA were retrospectively analyzed and informed and written consent was obtained from all study participants. The study was approved by the Ethics Committee of the aforementioned hospitals. Trans‐thoracic echocardiogram was performed in all patients before the procedure.

### 2.2 Electrophysiological study

**Figure 1:**
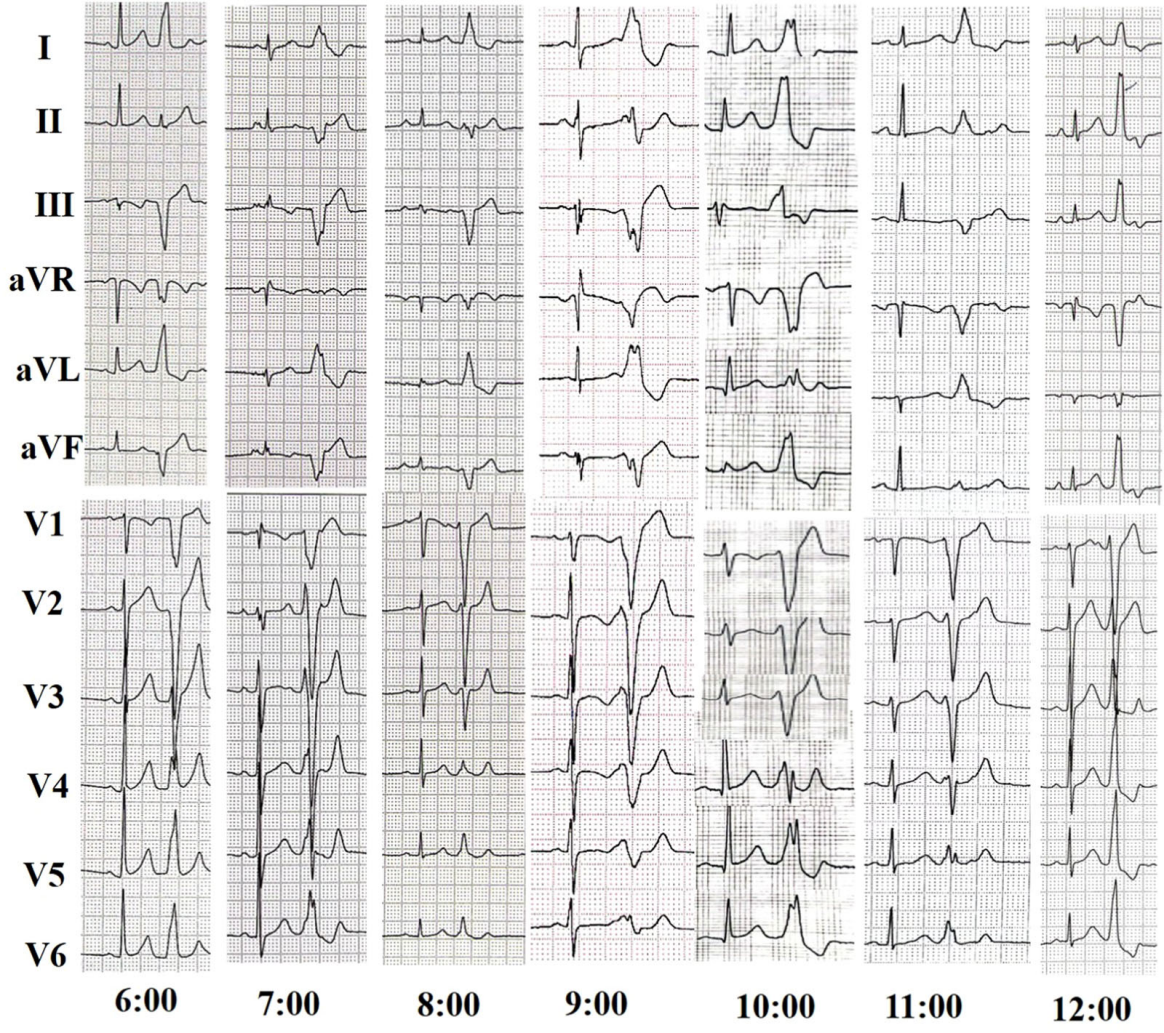
Representative 12‐lead electrocardiograms of idiopathic premature ventricular contraction originating from the free wall of tricuspid annulus at different sites.

All patients had their antiarrhythmic medication discontinued for at least five half‐lives before the start of the electrophysiological study. The study involved programmed electrical stimulation, as well as recordings of the 12‐lead surface electrocardiagram (ECG) (as shown in Figure 1) and intracardiac electrograms using the Lead 2000 electrophysiology system (Sichuan Jinjiang Electronic Science and Technology Co., Ltd.). In cases where the PVCs were not spontaneous or infrequent, isoproterenol infusion was administered intravenously to induce them. PVCs originating from the free wall of TA were defined as those where the earliest activation site was located in the free wall site of TA after complete mapping.

### 2.3 Mapping and catheter ablation

All procedures were performed by senior operators with more than 10‐year experience in cardiac ablation treatment. During the catheter ablation procedure, all patients underwent three‐dimensional electroanatomic mapping using either the Carto 3 system by Biosense Webster or the NavX system by St. Jude Medical. The catheter was used to map both supravalvular and subvalvular regions, and an 8.5‐F steerable sheath or nonsteerable sheath was utilized to facilitate catheter manipulation. A 7‐F 4‐mm‐tip irrigated catheter, including Navistar ThermoCool, SmartTouch by Biosense Webster, and Flexibility by St. Jude Medical, was used for mapping and ablation.

**Figure 2:**
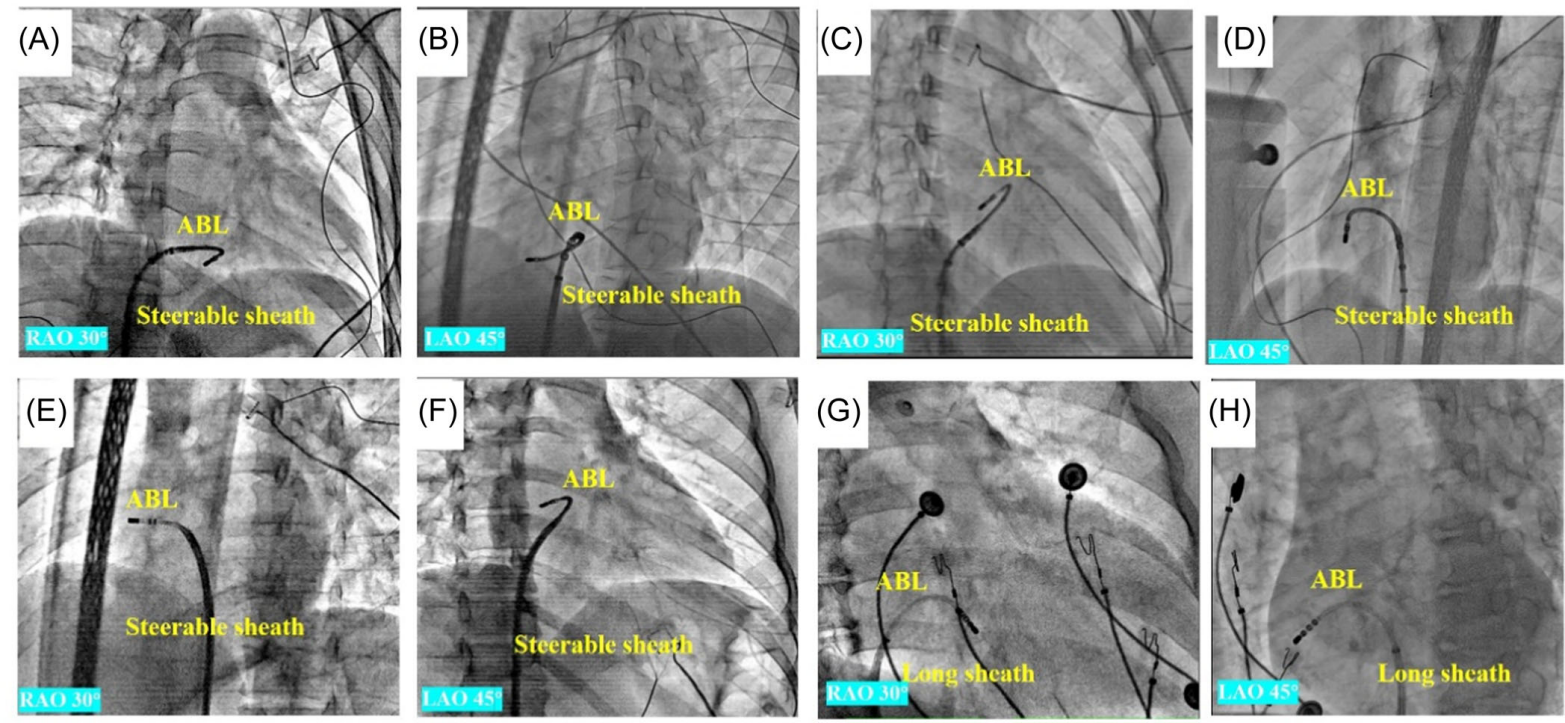
X‐ray image of ablation targets in RST group (A–F) and RCT group (G, H). (A, B) X‐ray image of the ablation used reversed S‐curve catheter technique target at 7 o'clock at RAO 30° and LAO 45° view, respectively. (C, D) X‐ray image of the ablation target used the reversed S‐curve catheter technique under the support of a steerable sheath at 9 o'clock at RAO 30° and LAO 45° view, respectively. (E, F) X‐ray image of the ablation target used the reversed S‐curve catheter technique under the support of a steerable sheath at 11 o'clock at RAO 30° and LAO 45° view, respectively. (G, H) X‐ray image of the ablation target used the reversed C‐curve catheter technique under the support of a nonsteerable sheath at 8 o'clock at RAO 30° and LAO 45° view, respectively. ABL, ablation catheter; LAO, left anterior oblique; RAO, right anterior oblique; RCT, reversed C‐curve technique; RST, reversed S‐curve technique.

The conventional method used a long sheath to facilitate the manipulation of the ablation catheter, which was advanced into the right ventricular (RV). After reaching the RV, the catheter was flexed and then withdrawn with a counterclockwise rotation toward the right atrioventricular annulus, where the amplitudes of the A and V waves were approximately equal. Subsequently, it was reached at the subvalvular region of the free wall for further examination. For the novel technique, the RST involved bending the distal end of the steerable sheath to a reversed C‐curve toward the septum and delivering the ablation catheter into the sheath. After reaching the RV, the ablation catheter was pulled back with counterclockwise rotation to the free wall of TA. This resulted in a reversed S‐curve shape of the ablation catheter in the RAO 30° view, hence the name RST (as shown in Figure 2).

Once the TA was reached, the ablation catheter showed a small A wave and a large V wave, with a ratio of A/V wave less than 1. If there were few spontaneous PVCs even after isoproterenol infusion, pacing mapping was performed to confirm the ablation site (Figure S1). Activation mapping was also conducted to identify the origin of PVCs. Additionally, the catheter and the ablated target were checked under fluoroscopy.

**Figure 3:**
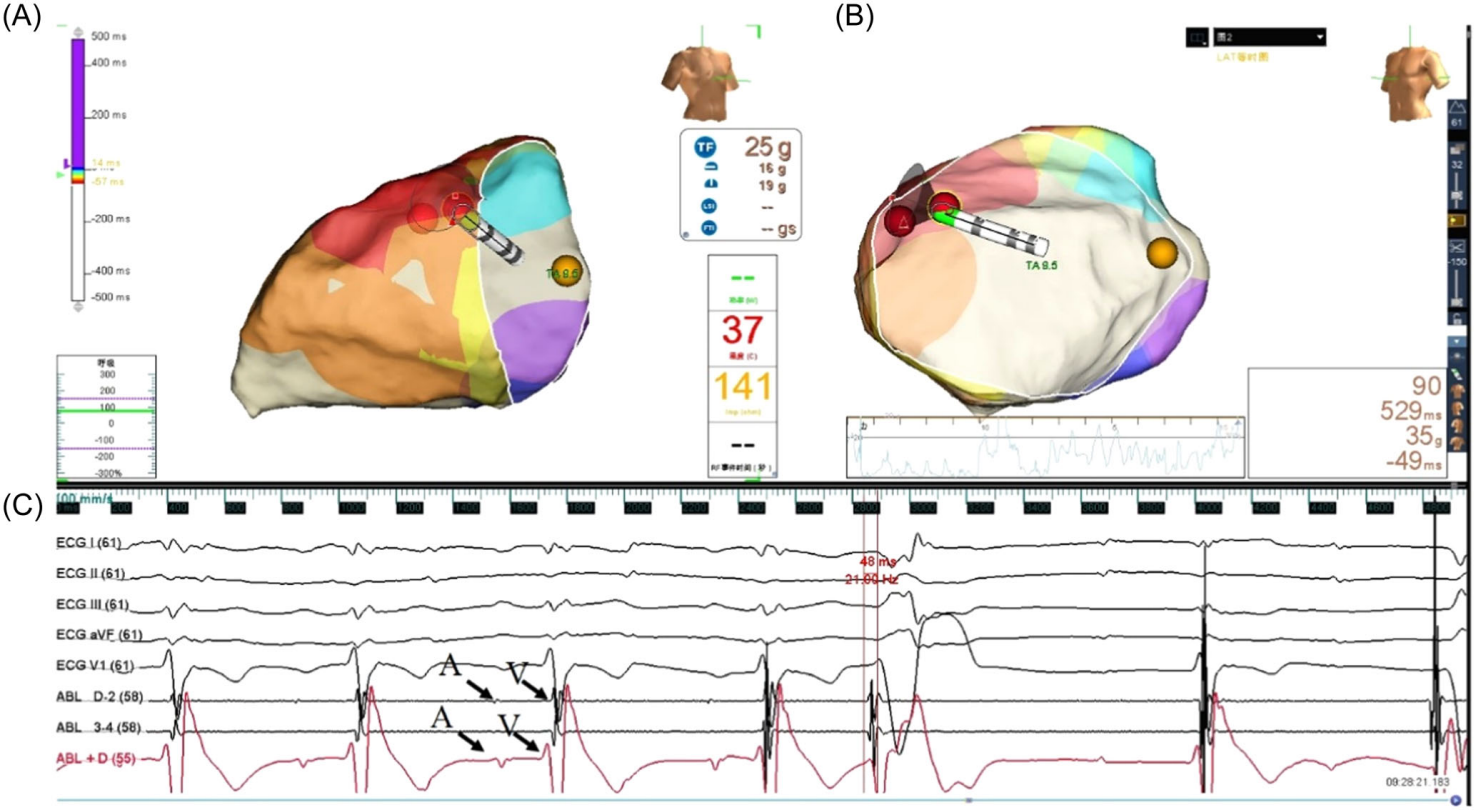
Successful ablation of PVCs originating from the free wall of TA with reversed S‐curve technique in another case at 11 o'clock of TA. (A) The view of successful ablation target site at RAO 30. (B) The view of successful ablation target site at LAO 45. (C) Earliest activation at the successful ablation site preceding the onset of PVC with 48 ms. The catheter showed a small A wave and a large V wave. LAO, left anterior oblique; PVC, premature ventricular contraction; RAO, right anterior oblique; TA, tricuspid annulus.

An irrigated catheter was performed for ablation with a maximum temperature of 43°C, a maximum power of 35 W, and an irrigated flow rate of 17 mL/min. The earliest activation recorded during the PVCs was considered the ablation target. If successful ablation was achieved within the first 10 s, radiofrequency (RF) energy was continued for an additional 90–120 s (Figure 3).

During the follow‐up, postablation PVC burdens were initially monitored using 24/48‐h Holter recordings within the first 3 months following the initial procedure. Subsequent follow‐up assessments and quantitative measurements of PVC burden were determined based on clinical judgment, considering factors such as symptoms, and the presence of coexisting arrhythmias. Acute failure was defined as the recurrence of PVC within 24 h postablation. The study used three criteria to determine successful catheter ablation: (1) absence of spontaneous PVCs or inducible PVCs after isoproterenol infusion, with at least 30 min of ECG monitoring at the end of the procedure; (3) no PVCs were observed during a 48‐h ECG monitoring period without antiarrhythmic drugs; (3) no recurrence of symptomatic PVCs was observed without antiarrhythmic drugs during at least 6 months of follow‐up.

### 2.4 Statistical analysis

The data are reported as mean ± standard deviation. Categorical variables are presented as absolute values and percentages. Statistical analyses were performed using SPSS 20.0 (SPSS Inc.). The Mann–Whitney test was used to compare non‐normally distributed variables between groups, while the Fisher's exact test was used for categorical variables. A *p* value of less than .05 was considered statistically significant.

## 3 RESULTS

### 3.1 Clinical characteristics

Table 1 presents the clinical characteristics of the two groups. Between January 2013 and November 2021, 59 patients underwent RF ablation of idiopathic PVCs originating from the free wall of TA. The RST group consisted of 26 patients, including 12 males (12/26) and 14 females (14/26), with an average age of 32.6 ± 11.7 years old. The RCT group consisted of 33 patients, including 16 males (16/33) and 17 females (17/33), with an average age of 34.9 ± 9.8 years old. One patient in the RST group was found to have an atrial septal defect (ASD), and one patient was diagnosed with dilated cardiomyopathy (DCM). In the RCT group, two patients were found to have ASD, and one patient was diagnosed with DCM. There were no significant differences in right atrium diameter, right ventricular diameter, left ventricular diameter, left ventricular ejection fraction (LVEF), palpitation duration, and PVC counts between the two groups (all *p* > .05).

**Table 1:** Comparison of clinical characteristics between both groups.

	RST group (*n* = 26)	RCT group (*n* = 33)	*p* Value
Male gender (*n*, %)	12 (46.2%)	16 (48.5%)	.9999
Age (years)	32.6 ± 11.7	34.9 ± 9.8	.4189
Right atrial diameter (mm)	29.8 ± 3.2	28.3 ± 3.3	.0725
Right ventricular diameter (mm)	30.1 ± 3.6	29.9 ± 2.6	.9423
Left ventricular diameter (mm)	46.7 ± 3.0	47.9 ± 1.9	.1302
LVEF (%)	59.6 ± 6.1	61.7 ± 5.3	.2093
Structure diseases (*n*, %)	2 (7.7%)	3 (9.1%)	.9999
ASD (*n*, %)	1 (3.8%)	2 (6.1%)	.9999
DCM (*n*, %)	1 (3.8%)	1 (3.0%)	.9999
Palpitation (months)	14.4 ± 10.2	14.0 ± 7.0	.8461
PVC count (no./24 h)	29 888.3 ± 7641.4	27 231.7 ± 5770.4	.2387

Abbreviations: ASD, atrial septal defect; DCM, dilated cardiomyopathy; LVEF, left ventricular ejection fraction; PVC, premature ventricular contraction; RST, reversed S‐curve technique.

### 3.2 Electrophysiologic characteristics and results of catheter ablation of PVCs arising from the free wall of TA between the two groups

The total procedural time was significantly shorter in the RST group compared to the RCT group (123.4 ± 18.8 vs. 146.3 ± 24.9 min, *p* = .0002), as shown in detail in Table 2. In addition, the fluoroscopic exposure time was significantly shorter in the RST group compared to the RCT group (16.9 ± 5.6 vs. 33.4 ± 5.2 min, *p* < .0001). There were no significant differences between the two groups in terms of mapping rhythm, earliest activation time, locations, and QRS waves duration (all *p* > .05). However, the success rate was significantly higher in the RST group compared to the RCT group (81.9% vs. 100%, *p* = .0297). Three patients in the RCT group failed to have successful ablation during the initial procedure and were successfully ablated using the novel method. During regular follow‐up, there were no recurrences in the RST group, while three patients in the RCT group experienced recurrence (*p* = .2748). The RST group demonstrated a significantly lower radiation dose compared to the RCT group (33.7 ± 7.3 Gycm^2^ vs. 55.3 + 7.4 Gycm^2^, *p* < .0001). Additionally, the RST group had a significantly lower duration of RF application compared to the RCT group (291.5 ± 71.2 s vs. 362.7 ± 112.6 s, *p* = .007). Moreover, the RST group exhibited a significantly higher contact force compared to the RCT group (12.0 ± 2.6 g vs. 9.9 ± 3.1, *p* = .003). Furthermore, the RST group had significantly fewer annotated ablation tags compared to the RCT group (8.1 ± 3.4 vs. 10.9 ± 3.2, *p* = .003).

**Table 2:** The electrophysiological procedure and catheter ablation characteristics of PVCs arising from the free wall of tricuspid annulus between the two groups.

	RST group (*n* = 26)	RCT group (*n* = 33)	*p* Value
Total procedural time (min)	123.4 ± 18.8	146.3 ± 24.9	.0002
Fluoroscopic exposure time (min)	16.9 ± 5.6	33.4 ± 5.2	<.0001
Radiation dose (Gycm^2^)	33.7 ± 7.3	55.3 + 7.4	<.0001
Pacing mapping (*n*, %)	3 (21.5%)	5 (15.1%)	.9999
Earliest activation time (ms)	41.2 ± 7.1	38.2 ± 7.8	.1761
QRS waves duration (ms)	150.5 ± 8.7	151.2 ± 11.2	.7878
Locations			
6:00–8:00	17 (65.4%)	18 (54.5%)	.4358
8:00–10:00	3 (11.5%)	6 (18.2%)	.7176
10:00–12:00	6 (23.1%)	9 (27.3%)	.7711
Complications (*n*, %)	0 (0%)	2 (6.1%)	.4985
Success rate (*n*, %)	26 (100%)	27 (81.9%)	.0297
Acute failure rate (*n*, %)	0 (0%)	3 (9.1%)	.2478
Recurrence (*n*, %)	0 (0%)	3 (9.1%)	.2478
RF application (s)	291.5 ± 71.2	362.7 ± 112.6	.007
Contact force (g)	12.0 ± 2.6	9.9 ± 3.1	.003
Noncontact force ablation (%)	2/26 (11.2%)	3/33 (9.1%)	.9999
Ablation temperature (°C)	40.4 ± 1.6	40.0 ± 3.0	.203
No. annotated ablation tags	8.1 ± 3.4	10.9 ± 3.2	.004

Abbreviations: PVC, premature ventricular contraction; RF, radiofrequency; RST, reversed S‐curve technique.

## 4 DISCUSSION

### 4.1 Major findings

The major findings of this study are as follows: First, the novel ablation method was associated with significantly shorter total procedural time and fluoroscopic exposure time compared to the conventional ablation method. Second, the success rate was significantly higher in the RST group than in the RCT group, and the novel method successfully ablated three patients who had failed with the conventional method. Finally, the RST group had no reported complications, whereas the RCT group had two cases of cardiac tamponade. These results suggest that the novel technique may be a more favorable approach for catheter ablation of PVCs originating from the free wall of TA.

### 4.2 PVCs originating from the TA

Idiopathic PVCs are typically characterized on ECG based on their anatomical origin.^8^, ^9^, ^10^ While PVCs can arise from any region of the TA, they are more commonly found to originate from the anteroseptal or para‐Hisian region rather than the free wall. Tada et al. conducted a detailed analysis of characteristic ECG features.^5^ As the TA is located on the right anterior side of the heart, all PVCs originating from the TA would present with a left bundle branch block QRS morphology and positive QRS polarity in inferior leads I, V5, and V6. When compared with PVCs originating from the right ventricular outflow tract, PVCs arising from the TA usually have an rS or QS pattern in lead augmented vector right, and a positive QRS polarity in lead augmented vector left. Late precordial transition is usually indicative of origin from the free wall, allowing for distinguishing between origin from septum or free wall of TA. QRS notching and waves duration are useful for distinguishing between PVCs originating from the septum or free wall of TA, with PVCs arising from the free wall having higher QRS notch and wider QRS waves duration. In this study, it was found that most TA PVCs arising from the free wall of TA originated at the 6:00–8:00 position, consistent with previous reports^5^ (details in Figure S2). PVCs originating from the lateral wall had a later precordial transition, wider QRS waves duration, and a higher QRS notch than those originating from the anterior or posterior wall.

### 4.3 Challenging in catheter ablation of PVCs originating from free wall of TA

Catheter ablation of PVCs is generally safe and has a high success rate, but can be challenging due to anatomical obstacles.^11^ Traditionally, PVCs originating from the TA were ablated using a supravalvular approach from the right atrium.^12^ However, when mapping the TA from the right atrium, the TA is often inadvertently mapped on the tricuspid valve, preventing effective RF delivery and resulting in low success rates and high recurrence rates.^13^ Poor mapping precision is another predominant reason for ablation failure. Recently, a subvalvular retrograde approach has been used for ablation of PVCs originating from the TA, which carries a higher acute success rate and lower recurrence rate than the conventional strategy.^14^ However, PVCs originating from the TA still pose a challenge due to catheter instability and poor tissue contact in the subtricuspid retrograde approach. The opening and closing of the valve can cause catheter instability, preventing effective RF delivery to the TA substrate of PVCs. In this case, high RF energy settings are recommended for ablation, but this increases the risk of right ventricular perforation, especially at the sites of TA free wall. Therefore, the ablation of PVCs originating from the free wall of TA remains a challenge.

### 4.4 Reversed S‐curve ablation technique with the support from a steerable sheath

To overcome catheter instability and poor tissue contact, the use of long guiding sheaths has been recommended for mapping and ablating PVCs originating from TA.^12^ If mapping or ablation fails, a large curve ablation catheter, including reversed C‐curve or U‐curve, should be applied for subvalvular region retrograde approach in the ablation of PVCs originating from para‐Hisian region.^15^, ^16^ These methods are feasible in patients with PVCs originating from the superior septal region due to the narrow space between the septal cusps of the TA and the septal wall of the right ventricle. However, there is little experience reported for PVCs originating from the free wall of TA. In this study, we report our experience of ablation of PVCs from the free wall of TA using a reversed S‐curve ablation technique with support from a steerable sheath. A stable contact force contributes to more effective lesions, long‐term successful outcomes, and a lower risk of complications. The use of a steerable sheath can improve catheter stability. Recent reports have shown that the use of a steerable sheath not only improves catheter stability but also reduces the procedural time and amount of energy.^17^ In our study, all patients were successfully ablated, and none experienced recurrence during long‐term follow‐up. No complications occurred during the procedure. In this retrospective study, both total procedural time and fluoroscopic exposure time were significantly shorter in the RST group compared to the RCT group. Patients in the RST group had a lower radiation dose, and higher success rate when compared to the patients in the RCT group. It indicated that the reserved S‐curve with the steerable sheath was much more stable than the Swartz sheath with Swartz sheath. Therefore, we used RST in the ablation of these PVCs in recent ablation.

In this study, we observed that the contacted force in RST group was significantly higher than that in RCT group. Contact force is a critical technical parameter that can significantly impact the success and safety of the procedure. If contact force is insufficient, the catheter may not make adequate contact with the tissue, resulting in incomplete ablation. This can lead to unsuccessful treatment, with PVCs persisting after the procedure. Incomplete ablation increases the risk of PVC recurrence, necessitating further procedures and potentially exposing the patient to additional risks and discomfort. Inadequate and excessive contact force can increase the risk of catheter perforation, where the catheter breaches the heart wall. This is a severe complication requiring immediate intervention. There was no significant difference between two group. However, the success rate was higher in RST group than in RCT group.

### 4.5 Clinical implications

Catheter ablation of PVCs deriving from the free wall is a challenging task and has a risk of right ventricular perforation. Herein, we introduced an RST under the support of a steerable sheath to overcome the anatomic obstacle because of the tricuspid valve movement. This novel technique supported a stable catheter contact and decreased the recurrent rate. It also can decrease the risk of right ventricular perforation.

### 4.6 Limitation

The sample size of patients with PVCs originating from the free wall of TA is small, which may limit the generalizability of the findings. The study was conducted in only three centers, which may limit the diversity of patient populations and treatment approaches. Therefore, additional studies with larger sample sizes and involving more centers are needed to confirm the findings and generalize the results.

## 5 CONCLUSION

Catheter ablation for PVC from free wall of TV via an RST under the support of a steerable sheath is effective and safe strategy.

## CONFLICT OF INTEREST STATEMENT

The authors declare no conflict of interest.

## Supporting information

Supporting information.

## Data Availability

The datasets used and/or analyzed during the current study are available from the corresponding author on reasonable request.
