# Supplementary material for: The comparison between the novel technique and conventional method in the catheter ablation of premature ventricular contractions originating from the free wall of tricuspid annulus
Source: Clin Cardiol. 2023 Oct 25;47(1):e24179. doi: 10.1002/clc.24179 (PMC10766136; doi:10.1002/clc.24179)

**Supplemental figures and figure legend**

**Supplemental Figure 1** Successful ablation of idiopathic PVCs originating from the free wall of TA with reversed S-curve technique under the steerable sheath in one case. A: 12 lead ECG showed the spontaneous PVC and sinus rhythm. Ablation catheter showed an A wave and V wave, the ratio of A/V <1. It indicated that the ablation catheter was located in the tricuspid annulus. B: Pace-mapping at the site of earliest activation. The pacing PVCs were almost the same with spontaneous PVCs. Pace mapping match score is 98%. C: Right anterior oblique views 30 of ablation catheter at the successful ablation site under X-ray. ABL = ablation; CS = coronary sinus; PVC = premature ventricular contraction.


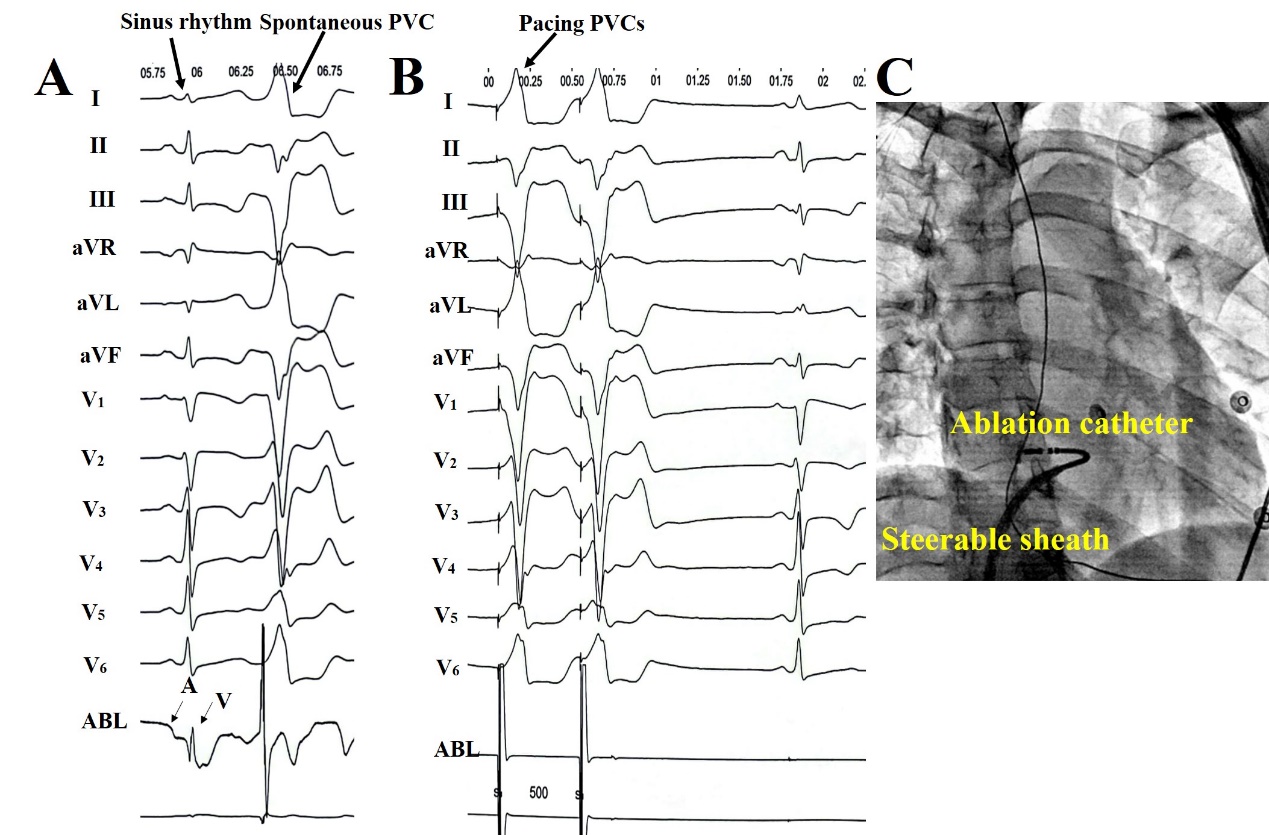


**Supplemental Figure 2**  The scheme of the distribution of PVCs originating from the free wall of TA in two groups. A: In RST group, there were 20 patients originating from 6:00 to 8:00 of TA, 7 patients from 8:00 to 10:00, and 9 patients from 10:00 to 12:00. B: In RCT group, there were 17 patients originating from 6:00 to 8:00 of TA, 3 patients from 8:00 to 10:00, and 6 patients from 10:00 to 12:00.


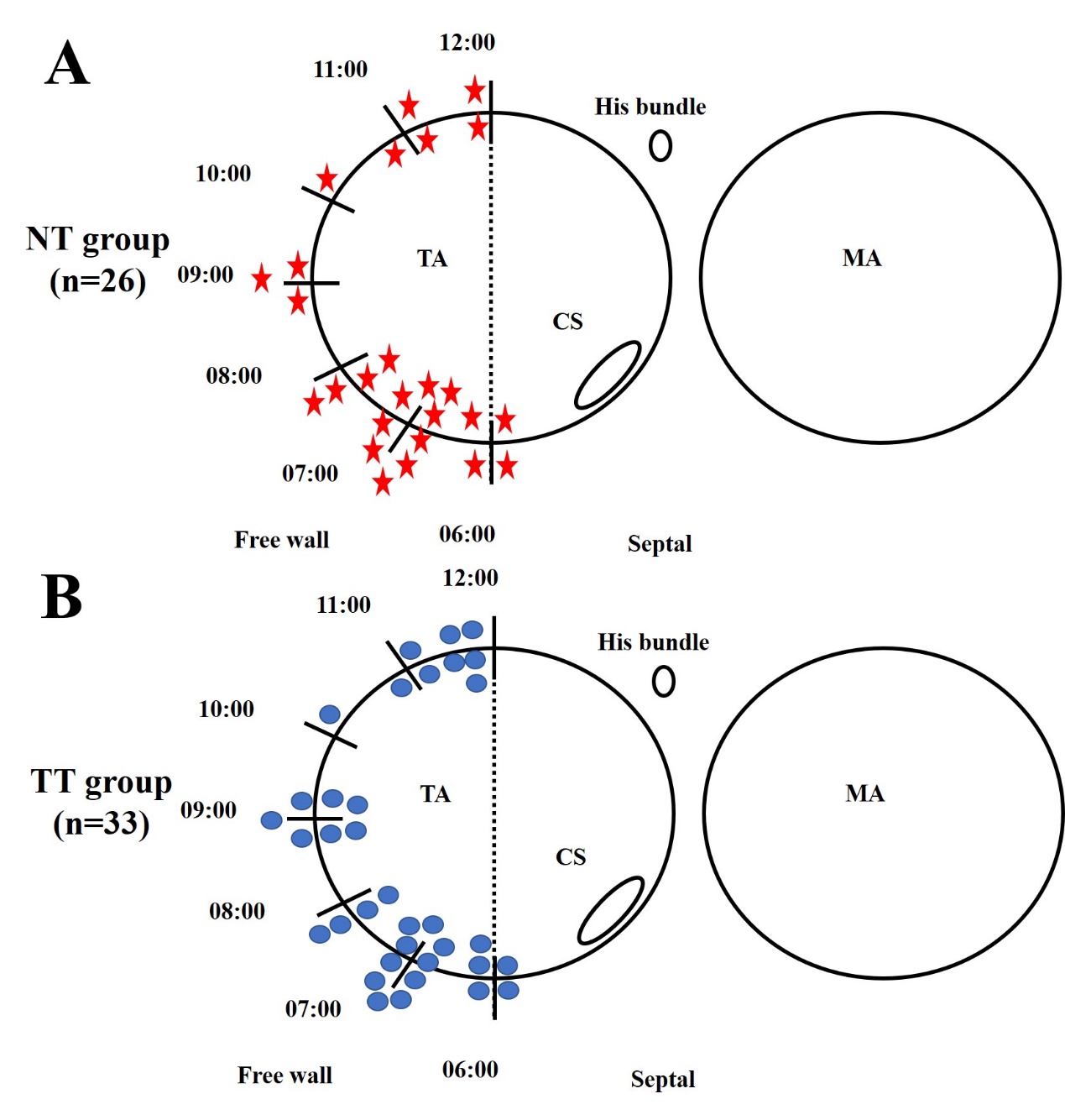

Supplement: Supplementary file 1 — Supporting information. [file CLC-47-e24179-s001.docx]
